# The transcriptome sequencing analysis reveals immune mechanisms of soybean fermented powder on the loach (*Misgurnus anguillicaudatus*) in response to Lipopolysaccharide (LPS) infection

**DOI:** 10.3389/fimmu.2023.1247038

**Published:** 2023-08-18

**Authors:** Yayuan Xu, Xinxin Zhang, Dongqi Li, Kun Qian, Yu Liu, Tingjuan Xu, Lishang Dai, Jianghua Cheng

**Affiliations:** ^1^ Institute of Agro-Products Processing, Anhui Academy of Agricultural Sciences, Hefei, China; ^2^ Anhui Engineering Laboratory of Food Microbial Fermentation and Functional Application, Hefei, China; ^3^ School of Pharmaceutical Sciences, Wenzhou Medical University, Wenzhou, China; ^4^ Gerontology Institute, The First Affiliated Hospital of University of Science and Technology of China (USTC), Division of Life Sciences and Medicine, University of Science and Technology of China, Hefei, China

**Keywords:** transcriptome sequencing, soybean fermentation powder, loach, LPS, immune function

## Abstract

The loach (*Misgurnus anguillicaudatus*), a small commercial fish that is widely cultivated for its high-quality protein, vitamins, minerals, and essential amino acid, is a member of the genus *Misgurnus* and the family Cyprinidae. In this study, we gave the LPS-injected loach fermented soybean meal and used transcriptome sequencing to investigate the impact of the fermented soybean powder on the loach’s immune system. 3384 up-regulated genes and 12116 down-regulated genes were found among the 15500 differentially expressed genes, according to the results. The differentially expressed genes were shown to be involved in cellular processes, metabolic processes, cellular anatomical entities, and binding, according to the Go functional annotation. Meanwhile, the KEGG enrichment analysis indicated that the soybean fermented powder treated groups showed significant differences in DNA replication, Nucleotide excision repair, Fanconi anemia pathway, and Base excision repair pathways, suggesting that these pathways are closely related to the enhancement of the immune function of loach by soybean fermented powder. The particular conclusions not exclusively can provide a new conception for the rational utilization of soybean fermented powder but also can provide theoretical guidance for the subsequent healthy breeding of loach.

## 1 Introduction

The loach (*Misgurnus anguillicaudatus*), a small commercial fish (1–3) that is widely cultivated for its high-quality protein, vitamins, minerals, and essential amino acid, is a member of the genus *Misgurnus* and the family Cyprinidae (4, 5). In traditional Chinese medicine, because of its nutritional and medicinal value, the loach is often called ‘Ginseng water’ (6). Furthermore, the loach is widely distributed in low-lying rivers in Asia (7, 8) and has become a traditional and popular food in East Asian countries such as China and Korea (8–11). The loach can smartly and flexibly inhabit the bottom mud and survive under extreme conditions such as rising water temperature and arid environments (12, 13), but diseases caused by microbial infection and other environmental factors have threatened the healthy growth of the loach (14, 15). Nevertheless, the use of antibiotics and pesticides is severely restricted in many countries (16, 17). Therefore, the spreading of the loach industry is bounded to a certain extent.

In the present circumstances, many studies have been carried out on the aquaculture, respiratory physiology, and lipid metabolism of the loach (18, 19). Studies have shown that the use of phenolic compounds as feed additives for farmed fish provides many beneficial effects on fish health and performance by strengthening the immune system and stimulating appetite, and improving intestinal digestion and mucosal function, morphology, and integrity, as well as gut microbial community composition (20–22). The fermented soybean dregs contain abundant active components, such as phenolic compounds (23, 24). As a consequence, we put forward a bold hypothesis: using soybean fermentative powder to feed loach can not only make rational use of resources; but also solve the problem of healthy growth of loach due to microbial infection and other environmental factors.

With the gradual development of sequencing technology and decreasing cost, transcriptome sequencing technology has been applied to plant breeding, stress, genetic analysis, etc. (25–27). Lipopolysaccharide, a pro-inflammatory compound from the outer walls of Gram-negative bacteria, promotes intestinal inflammation. A connection between low-grade inflammation, sustained by lipopolysaccharides (LPS), and the development of metabolic disorders is well established (28, 29). In this study, after treatment with LPS (Beijing Solarbio Science & Technology Co., Ltd.), then they were randomly divided into two groups, one group was fed with fermented soybean powder, and the other group was not. After nourishing for a certain, based on transcriptome sequencing technology, the related mechanism of soybean fermented powder improving the immune function of the loach was inspected, to provide theoretical guidance for the subsequently healthy breeding of the loach.

## 2 Materials and methods

### 2.1 Experimental animal and LPS treatment

The loaches were bought from the province of Zhejiang’s aquaculture base. In a lab setting, forty robust, healthy, and similar-sized loaches were chosen after fifteen days of domestication using dechlorinated tap water for three days. Their dimensions were 37 cm long, 31.5 cm wide, and 13.5 cm tall.

The forty loaches were randomly and equally split into two groups, one serving as the control group and the other as the group that received the soybean fermented powder treatment. Using sterile PBS (Biosharp Biotechnology Co., Ltd.) as the solvent, a suitable quantity of lipopolysaccharide (LPS) powder was weighed with an electronic balance. 100 ul of lipopolysaccharide solution should be injected into the rear muscle of the loach. Following that, they were given separate feedings both with and without soybean fermented powder. The surface of the loach was cleaned with 75% alcohol after it had been fed for 24 hours, and the loach was then put to sleep (MS222, Beijing Green Hungering Biotechnology Co., Ltd.). The muscles were cut into small pieces with sterile scissors and placed in centrifuge tubes without enzymes. Using the use of sterile scissors, the muscles were divided, placed in enzyme-free centrifuge tubes, immediately frozen using dry ice, and afterward kept at -80°C.

### 2.2 RNA extraction, library construction and sequencing

Using the TriZol method (Life Technologies), Total RNA from the loaches muscle was extracted according to the manufacturer’s requirements. The extracted RNA was analyzed by agarose gel electrophoresis, Nanodrop photometer, and Agilent 2100.

After passing the RNA quality evaluation, the mRNA with Polya tail was enriched by magnetic beads with Oligo (DT), and then the mRNA was interrupted by ultrasound. The first strand of cDNA was synthesized in an M-MuLV reverse transcriptase system using a fragmented mRNA template and a random oligonucleotide as a primer, followed by degradation of the RNA strand with RNaseH, and then in a DNA polymerase I system, the second strand of cDNA was synthesized from dNTPs. After the purified double-stranded cDNA was repaired, a-tailed, and connected with the sequencing linker, about 200 bp cDNA was screened with AMPure XP beads, and the PCR products were amplified by PCR and purified again with AMPure XP beads, finally getting the library.

### 2.3 *De novo* assembly and basic function annotation

Clean reads were obtained, by filtering low-quality data, to ensure the quality and reliability of data analysis. Then the reads are assembled, and the generated Unigenes are compared with the sequence of the public database to complete the functional annotation.

We compared Unigene sequences to NR (Non-Redundant Protein Sequence Database), SwissProt, KEGG (Kyoto Encyclopedia of Genes and Genome), KOG (Clusters of Orthologous Groups of proteins), and GO (Gene Ontology) databases by the Blast, and performed functional annotation

### 2.4 Analysis of differentially expressed genes

What seems necessary to identify differentially expressed genes from muscle samples is to study the effect of soybean ferment powder on the immune mechanism of the loach. The RPKM was used to calculate gene expression, as well as the DedgeR software was adapted to analyze the differential expression between the two groups. The genes with FDR < 0.05 and log2(FC) > log2(2) were considered as differentially expressed genes. On that occasion, the GO and KEGG enrichment analysis was performed to identify the biological functions and major metabolic pathways of differentially expressed genes.

### 2.5 qRT-PCR verification

The consequences of differentially expressed genes were verified by Quantitative real-time PCR (qRT-PCR). We selected seven up-regulated genes and eight down-regulated genes according to the principle of │log2(fc)│>1 and fpkm > 10. Primers for q-PCR were designed using the DNAstar software. The length of the primer was 18–22bp while the length of the product is 80-200 bp. The q-PCR was performed for the target genes from cDNA prepared using SYBR^®^ Green Supermix (TransGen Biotech). Data were analyzed using the comparative Ct (Cycle Threshold) quantification method which compares the relative expression of target genes to the expression of a reference gene. Delta-delta CT method was used to calculate relative gene expression levels in which Δct (target gene Ct-reference gene Ct), ΔΔct (Δct treatment-Δct control), and fold change (2^-ΔΔct^) were calculated (30–32), and β-actin was used as an internal reference gene (33) (**Table 1**).

**Table 1:** Primer sequences for qRT-PCR assay.

Gene Symbol	Forward primer (5′–3′)	Reverse primer (5′–3′)	Product size (bp)
ddit4	ACAGCGTGGACTCAGATTCAGA	GCCTCCTCGCTCGGGTCAAA	121
ACTA1	CAGCTTGCGGCGGAAATACTT	CCTGGCCCGTCTGATGAATG	161
LOC129425453	ATAGCACCACCGACAATCTTCC	TACTTTAGTCCCGCCCTCTGC	149
LOC129416000	CCGCGCCGATGTTCTTCT	TCATCAGGTACGGGCTTTCTTG	168
pfkfb4b	ACAAAACCCACTGCGGAAAATA	CAGGCAAACCCACAGTCACAAT	122
LOC129436072	TCGTGACTGTGAGGCTTTGAT	GCGTCCACCCCTCTTGTTCTG	199
clk2b	GTTTCCACCCGACATTACAG	ATACAGCCGATACTCCAGACAT	88
thy1	AACCGTGGCGCAGTCAAA	GCAGGATACACCAGGCAGAAC	178
mid1ip1l	AAGAGGGCCCGCAGCAGATA	ACAGAACCCCAAACAGTCCA	96
LOC107755690	ATCCCAAGCGAATCAAAGTGT	GTTCCCATCAGCCAGTTCCT	89
psme3	AAGCTGGTGGACCTGATTGA	TTGTTGCCGTCTTCTATTCTTG	116
h3f3a	CGCGCCCTCTACTGGTGGTGT	ACGCAGGTCGGTTTTGAAGTCC	166
abcf2a	AAGGCGGCAAAGAAGAAGGAAG	GAGATGCCAGCACACCAGTCAC	199
hbegfa	GGGGATTACGGTTTGCCATTAG	TTCCCTTTCCTTTCGTTTTCTT	130
nqo1	ATGGCGGGAAAGGGTGAAT	GATGATGTGCGGTGGAGATGC	158
β-actin	AGAGAGAAATTGTCCGTGAC	GCCAATGGTGATGACCTGT	140

The Forward primer is a primer that binds to the sense strand in the DNA double-strand and the Reverse primer a primer that binds to the sense strand.

## 3 Results and discussion

### 3.1 Data quality control and Trinity Assembly

By sequencing the control group (Group A) and the soybean fermented powder treatment group (Group B), 43,377,890 and 45,397,028 raw reads were obtained independently. Using the fastp (34) to control the quality of raw reads and filter the low-quality data, nevertheless, 43,190,774 (99.57%) and 45,079,698 (99.30%) clean reads were obtained one after the other. The more balanced the base composition, the higher the quality and the more accurate the subsequent analysis. We analyzed the composition and mass distribution of clean reads bases. The GC contents of group A and group B were 44.31% and 44.47% step by step, and Q20% (the percentage of bases with base recognition accuracy above 99%) was 97.14% and 96.46% separately, and Q30% (the proportion of bases with base recognition accuracy above 99.9%) was 91.67% respectively (**Table 2**).

**Table 2:** Summary of the sequence analyses.

Sample	Raw reads	Clean reads(%)	Q20(%)	Q30(%)	GC( %)
A	43377890	43190774(99.57)	97.14	91.67	44.31
B	45397028	45079698(99.30)	96.46	90.39	44.47

Raw reads: From whole-genome sequencing. Clean reads: The number of reads after removing low-quality sequences. The subsequent analysis is based on clean reads.Q20 and Q30: The percentage of bases with Phred values >20 and >30, respectively. GC: The GC proportion of filtered sequence bases.

**Figure 1:**
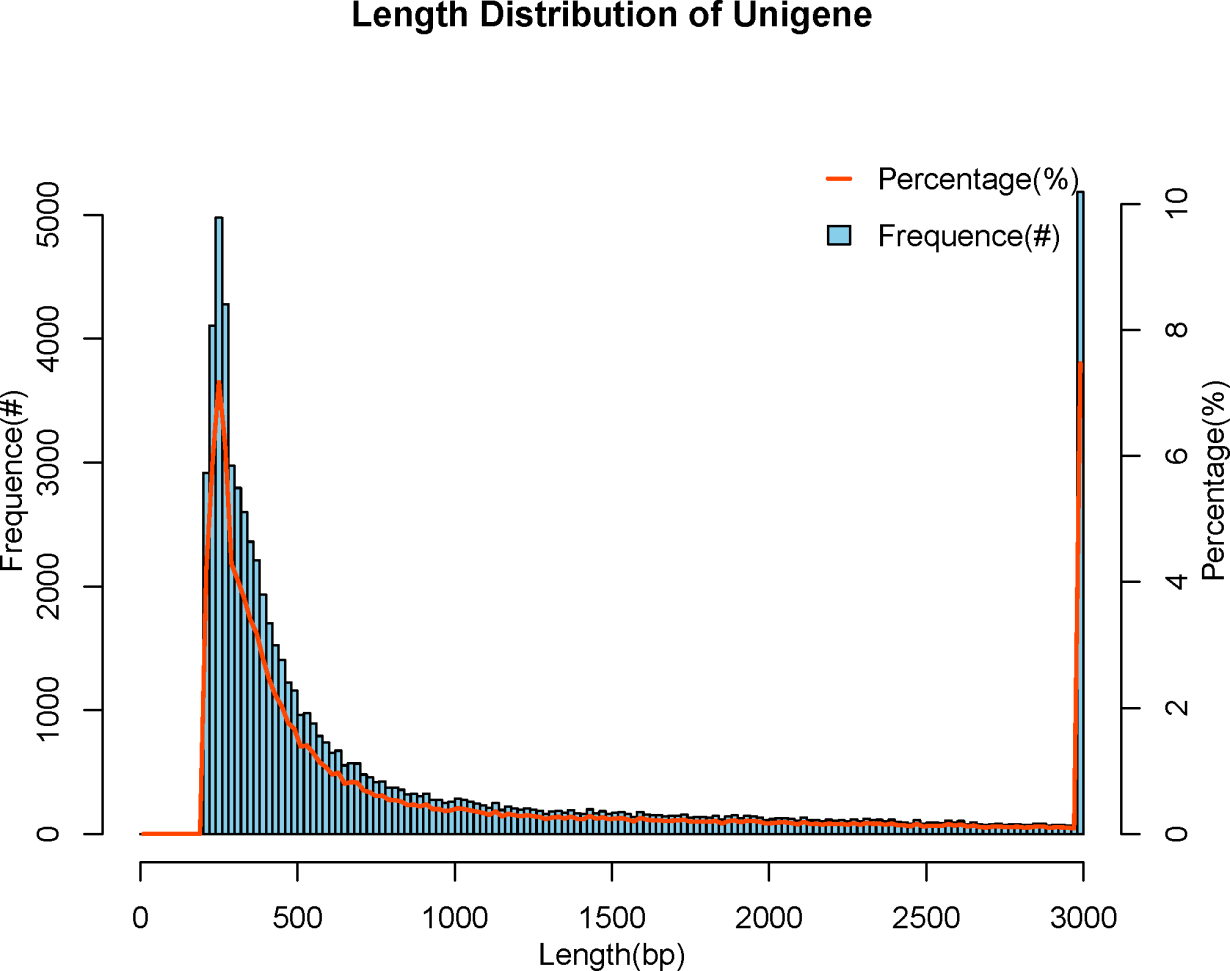
Length distribution of Unigene. The abscissa indicates the length of assembled Unigenes, and the ordinate indicates the number of Unigenes under the corresponding length.

We used Trinity Software to assemble reads (35) and adopted N50 numerical values to assess the quality of the assembly results, with longer N50 and fewer numbers indicating better assembly quality. All Unigenes are categorized from long to short, and the length is cumulated sequentially, corresponding to the length and number of that fragment when the cumulative fragment length reaches 50% of the total fragment length (the length of all Unigenes), that is the length and quantity of the Unigene N50. The number of the N50 was 9485, with the length being 2065 bp, in the meanwhile, the maximum length was 50577 bp and the minimum length was 201 bp, and the average length was 985 bp (**Table 3**). We attained a total of 69384 Unigenes. The length of the Unigenes was between 201 bp and 50577 bp, mainly distributed between 200 bp and 500 bp, and the rest were less distributed in the interval (**Figure 1**).

**Table 3:** Assembly quality statistics.

N50 number	N50 length	Max length	Min length	Average length
9485	2065	50577	201	985

N50 number: the number of Unigene N50. N50 length: The length of Unigene N50. Max length: Assemble the longest unigene length. Min length:Assemble the shortest unigene length. Average length: The average length of unigene is assembled.

### 3.2 Unigene sample assessment

We used the assembled Unigene as a reference and counted gene detection in samples from the control and treatment groups, with 47,914 genes detected in the control group and 40,793 genes detected in the treatment group.

**Figure 2:**
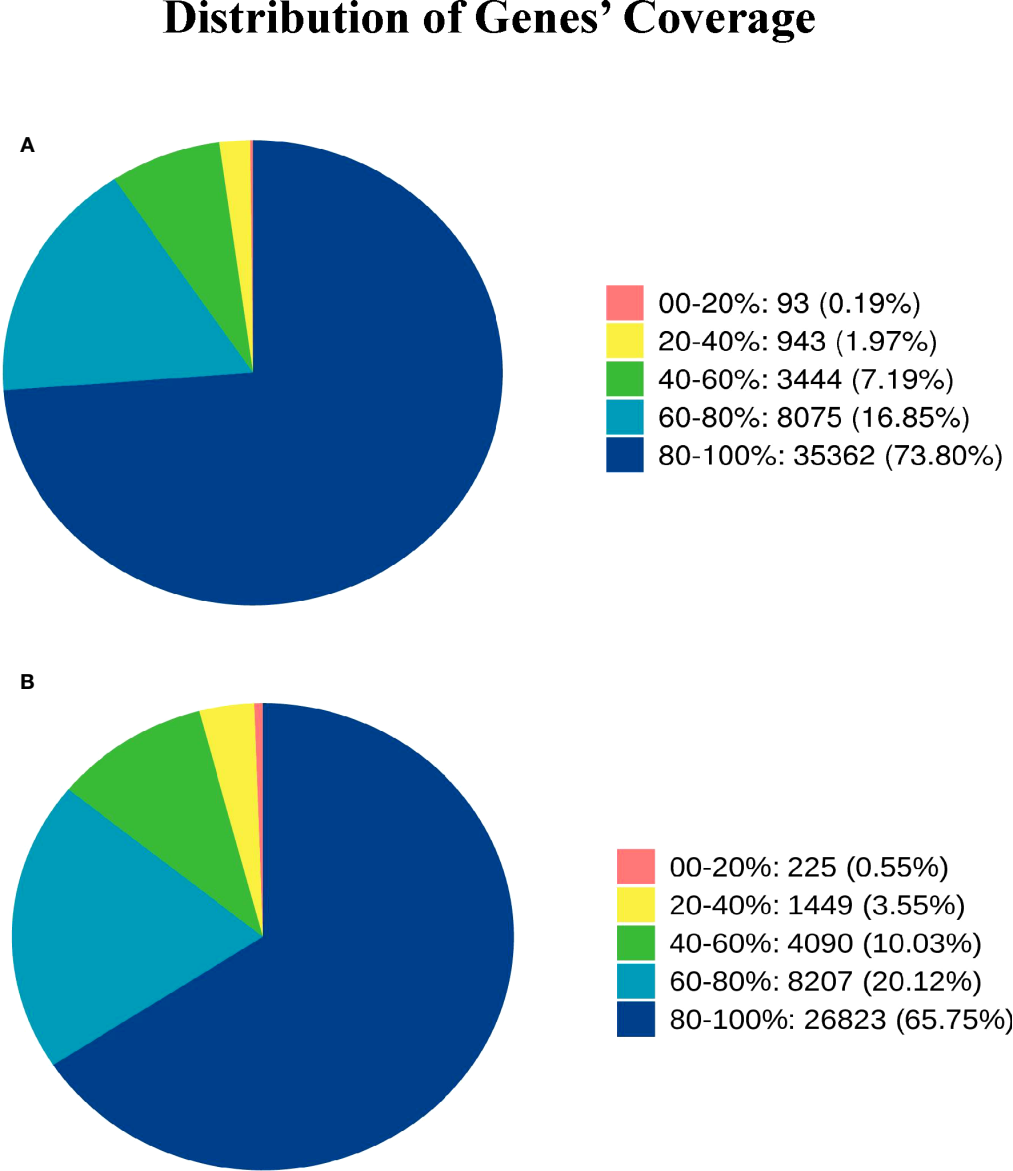
Distribution of Genes’ Coverage. Different colors in the graph represent the proportion of genes with a certain coverage range. **(A)** represents the gene coverage rate of Group A, and **(B)** represents the gene coverage rate of Group B.

Gene coverage is the percentage of each gene covered by reads, which is equal to the ratio of the number of bases covered by the reads in the gene to the number of bases in the gene coding region.The gene coverage of Group A and Group B is 80%-100%, accounting for 73.80% and 66.75% one after the other, and the gene coverage of 60%-80% accounts for 16.85% and 20.12% successively (**Figure 2**)

### 3.3 Unigene basic annotation

Aiming to evaluate the function of the obtained Unigenes, gene function annotations were performed on these Unigenes. We aligned Unigene sequences to NR (Non-Redundant Protein Sequence Database), SwissProt, KEGG (Kyoto Encyclopedia of Genes and Genome), and KOG (Clusters of Orthologous Groups of proteins) by Blast. The results showed that of the 69384 Unigene sequences, 30419 were annotated while 38965(56.16%) were unannotated. Of these, the NR database (43.58%) and KEGG database (42.33%) had the most annotations, followed by the SwissProt database (30.94%) and KOG database (24.00%) with the minimum annotations (**Table 4**).

**Table 4:** Annotation statistics of four major databases.

**Database**	**number**	**percentage(%)**
Nr	30237	43.58
KEGG	29373	42.33
KOG	16646	24
SwissProt	21465	30.94
Annotation genes	30419	43.84
Without annotation gene	38965	56.16

Database: Type of database. Number: Represents the number of unigenes successfully annotated in the database. Percentage (%): represents the proportion of unigenes successfully annotated in the database to the total unigenes. Annotation genes: Compare the number of unigene in the last four databases. Without annotation gene: The number of unigene that are not matched to the database.

#### 3.3.1 NR annotation

**Figure 3:**
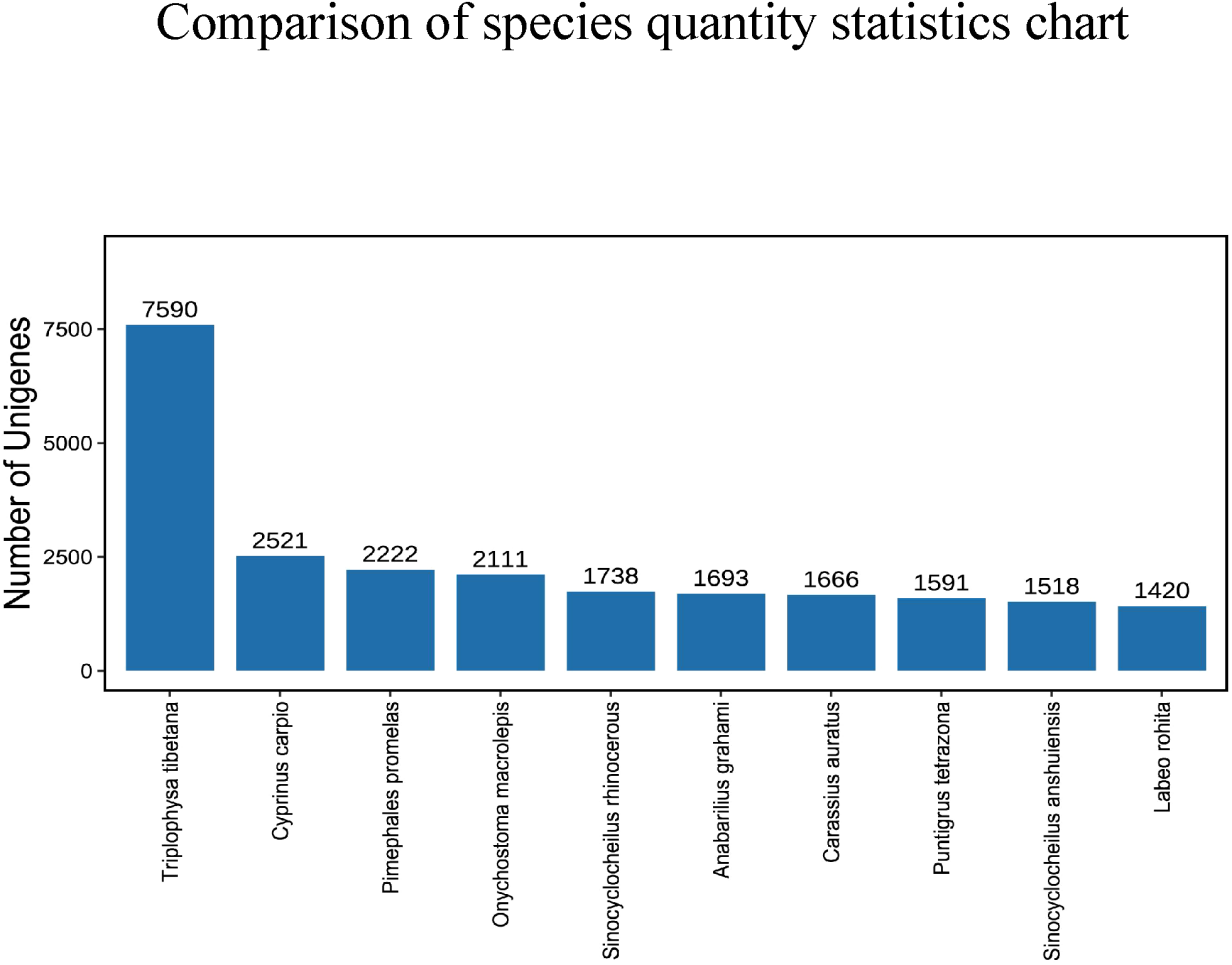
Comparison of species quantity statistics chart.

After comparing the Unigene sequence with NR (Non-Redundant Protein Sequence Database) database, the sequence with the best comparison result (the lowest e value) in the NR database is the corresponding homologous sequence (if there is juxtaposition, take the first one) to determine the species to which the homologous sequence belongs; and count the number of homologous sequences of each species. The consequences displayed that the amount of similarities between the Unigene sequence and *Triplophysa tibetana* was the highest, reaching 7590, followed by *Cyprinus carpio* and *Pimephales promelas*, with 2521 and 2222 respectively. The number similar to *Sinocyclocheilus anshuiensis* and *Labeo rohita* is the least, with 1518 and 1420 individually (**Figure 3**).

#### 3.3.2 KEGG annotation

**Figure 4:**
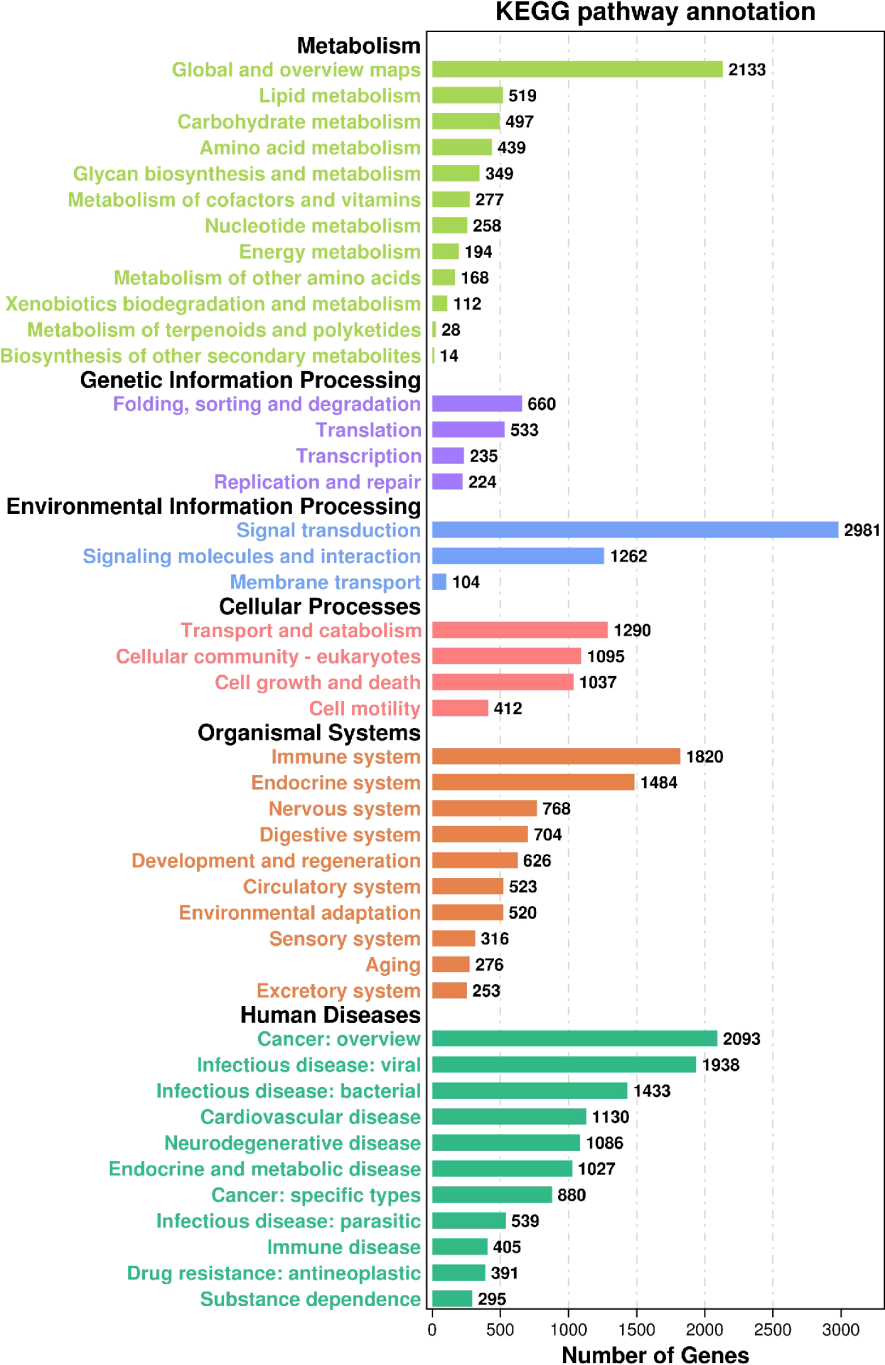
KEGG pathway annotation.

KEGG (36), (Kyoto Encyclopedia of Genes and Genomes), is a database that integrates genome, chemistry, and system function information, targeting revealing the genetic material and chemical blueprint of life phenomena. The KEGG annotation outcomes displayed that (**Figure 4**) a total of 29,373 Unigene were annotated to 359 paths. In Human Diseases, there exist 11,217 annotations (33.66%), and in Genetic Information Processing, there stand 1652 annotations (4.96%). In Human Diseases, the most annotated path is Cancer: overview, with 2093 entries, and the least annotated path is Substance dependence, with 295 entries. In Genetic Information Processing, the most annotated paths are Folding, sorting, and degradation, with 660 paths, while the least annotated paths are Replication and repair, with 224 paths. In the Metabolism class, 4,988 (14.97%) Unigenes are annotated the most, and the annotated paths are Global and overview maps, with 2,133, while in Metabolism of terpenoids and polyketides, and Biosynthesis of other secondary metabolites, with 28 and 14 annotations respectively. In the Environmental Information Processing, 4347 items (13.04%) are annotated, the most and least annotated paths are Signal transformation and Membrane transport respectively, 2981 Unigenes are annotated in the Signal transformation path, and 104 Unigenes are annotated in the Membrane transport path. In Cellular Processes, 3834 (11.50%) are annotated, the Transport and catabolism paths are annotated the most, there are 1290 Unigenes, and the Cell mobility paths are annotated the least, with 412 Unigenes. In Organismal Systems, 7290 Unigenes are annotated, in the meantime, the Immune system path is annotated the most, with 1820, and the exception system path is annotated the least, with 253.

#### 3.3.3 KOG annotation

**Figure 5:**
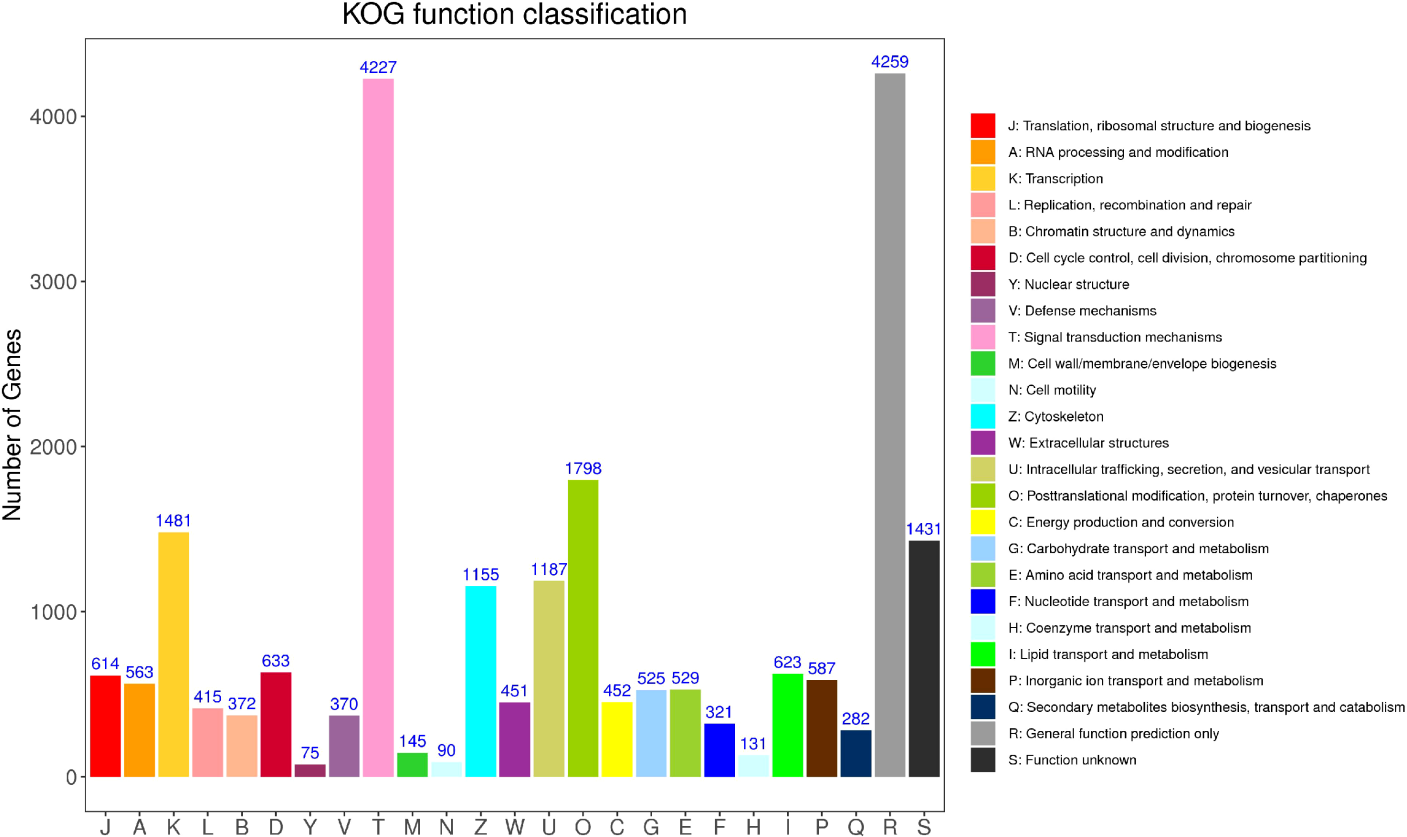
KOG classification statistical chart.

According to the annotation results of the COG (Clusters of Orthodoxy Groups of Proteins) database (**Figure 5**), there are 16,646 Unigene annotated to 25 types of functions. The top five categories with the predominant proportion are general function prediction only (18.75%), signal translation mechanisms (18.61%), post-translational modification, protein turnover, chaperones (7.92%),Transcription (6.52%), and Function unknown (6.30%). In addition, cell mobility (0.40%) and Nuclear structure (0.33%) have the smallest comments. What can be observed is that the proportion of unknown function classification is small, only 6.30%.

#### 3.3.4 GO annotation

**Figure 6:**
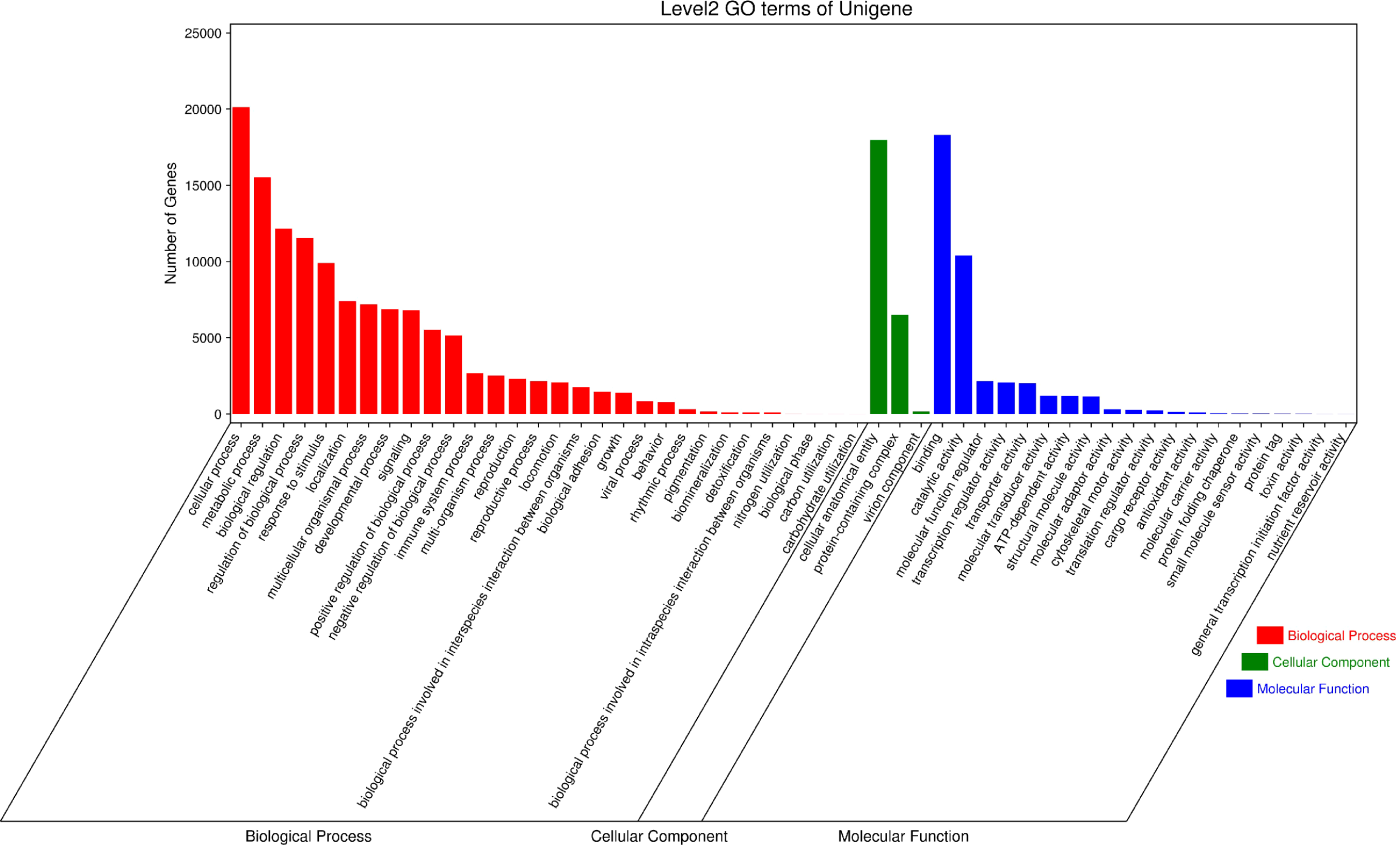
GO secondary classification statistical chart. The results are summarized into three ontologies (Cellular Component, Biological Function and Molecular Function).

GO (Gene Ontology) has three ontologies, which respectively describe the Molecular Function, Cellular Component, and Biological Process of genes. According to the annotation results of the GO database (**Figure 6**), 126,958 Unigenes are annotated in Biological Processes, 39,666 Unigenes are annotated in Molecular Function and 24,624 Unigenes are annotated in Cellular Components. In the Biological Process classification, the top three categories of secondary classification annotations are cellular process(20131), metabolic process(15537), and biological regulation(12159). In the classification of Molecular Function, the top three secondary annotations are binding(18304), catalytic activity(10409), and molecular function regulator (2,152). In the classification of Cellular Components, only three secondary classifications are annotated, namely cellular anatomical entity(17981), protein-containing complex(6504), and virion component (157).

### 3.4 Differential expression analysis

#### 3.4.1 Global analysis of differentially expressed genes

**Figure 7:**
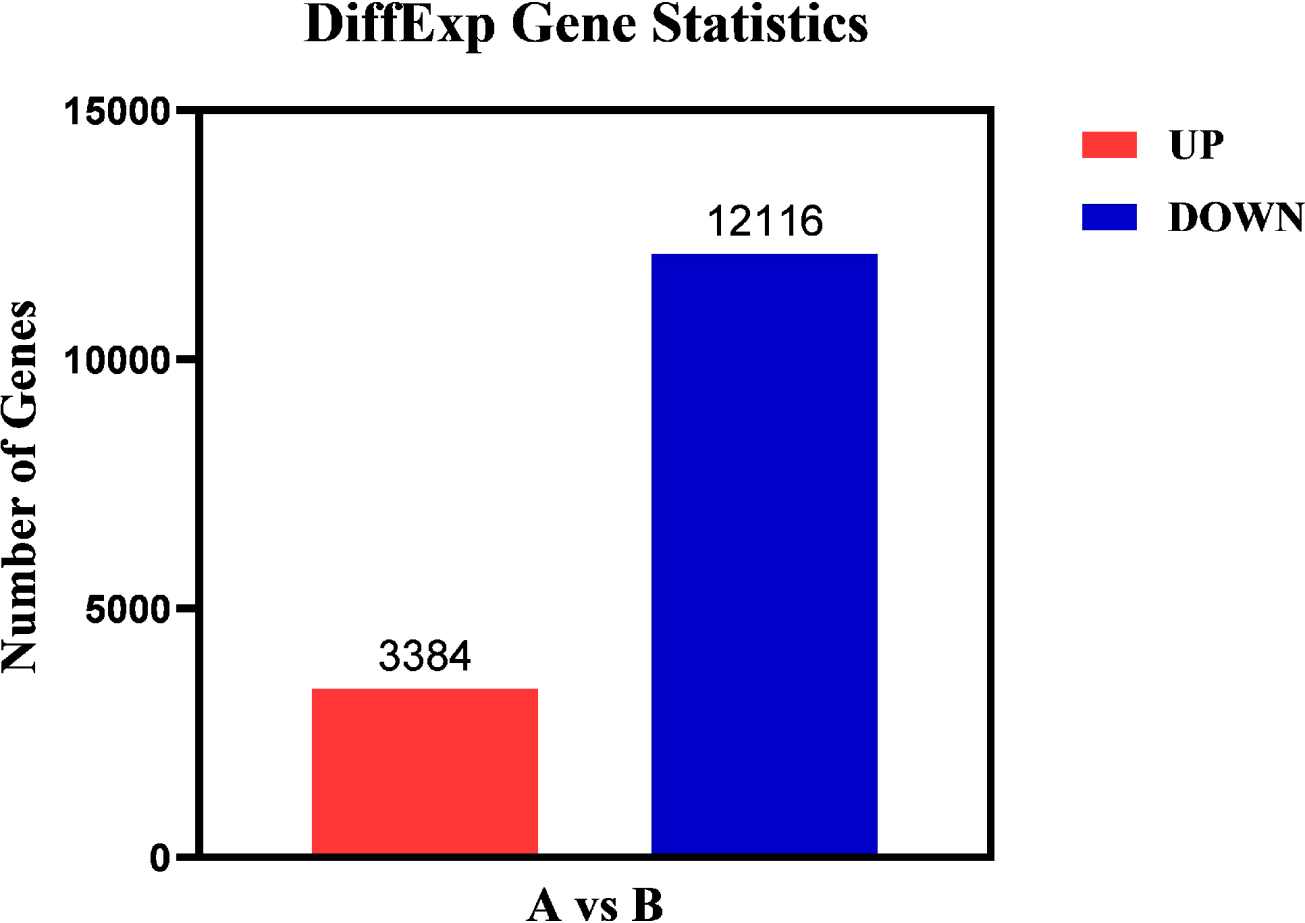
Differential gene statistical map. Abscissa: samples compared in pairs; Ordinate: the number of differentially expressed genes; Red represents up-regulated differential genes; Blue represents down-regulated differential genes.

The differentially expressed genes in Group A and Group B were analyzed by edgeR (37) software, and the differential screening conditions were: FDR<0.05, |log2FC| > log2 (2). Compared with Group A, the total quantity of differentially expressed genes in Group B was 15,500, with 3,384 genes up-regulated and 12,116 genes down-regulated (**Figure 7**).

**Figure 8:**
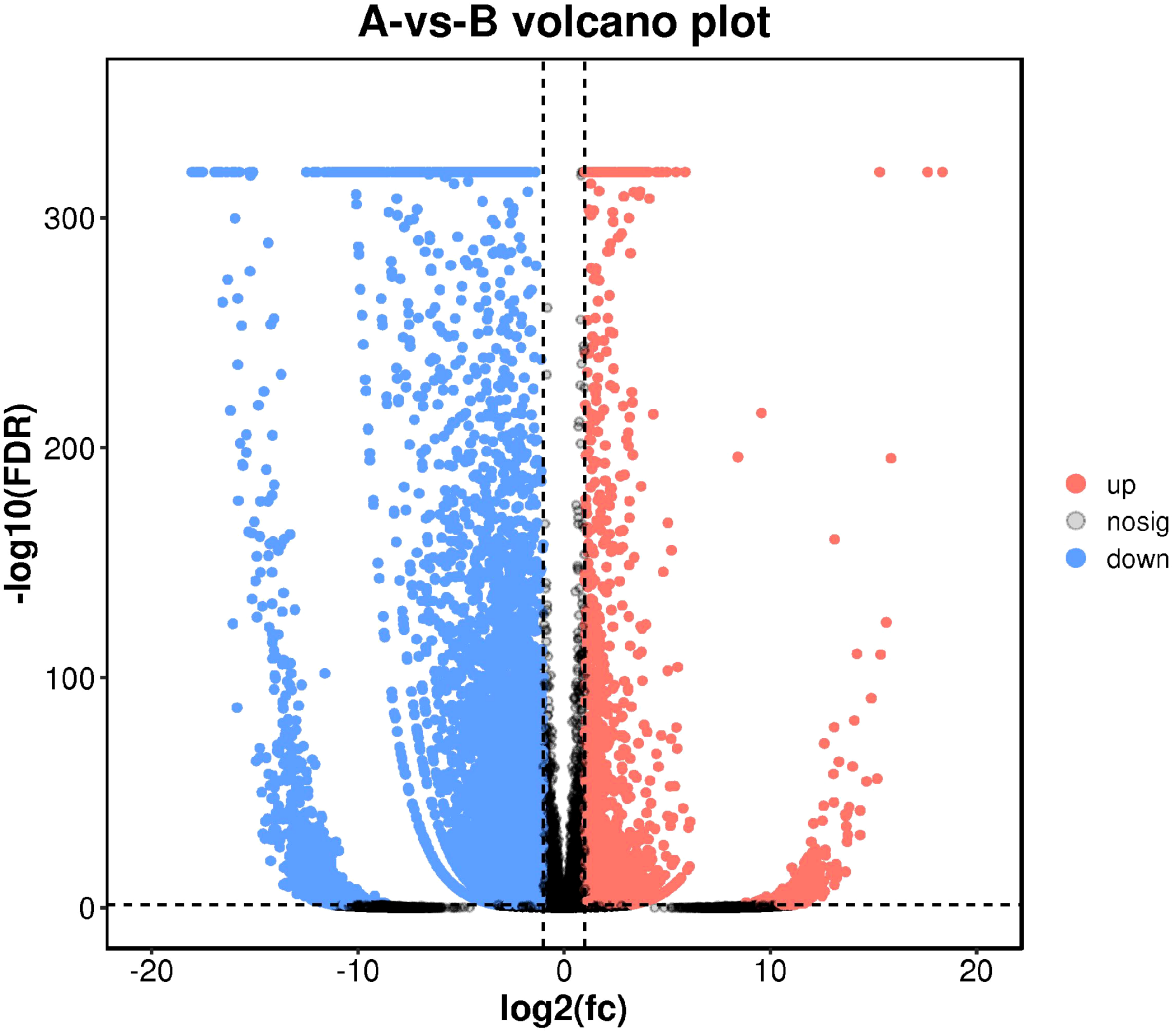
Differential comparison volcano map. The abscissa represents the logarithmic value of the difference multiple between the two groups, and the ordinate represents the negative Log10 value of the difference FDR between the two groups. The red (up-regulated expression of Group B relative to Group A) and blue (down-regulated expression) dots indicate that the gene expression is different (the judging standard is FDR <0.05, and the difference multiple is more than twice), and the black dots indicate that there is no difference.

As maintained by the significantly different genes in each comparison group, we carried out a volcanic map analysis. The volcano map can visually show the distinct expression genes between Group A and Group B. The closer to the genes at both ends, the grander the difference (**Figure 8**).

#### 3.4.2 GO enrichment analysis

**Figure 9:**
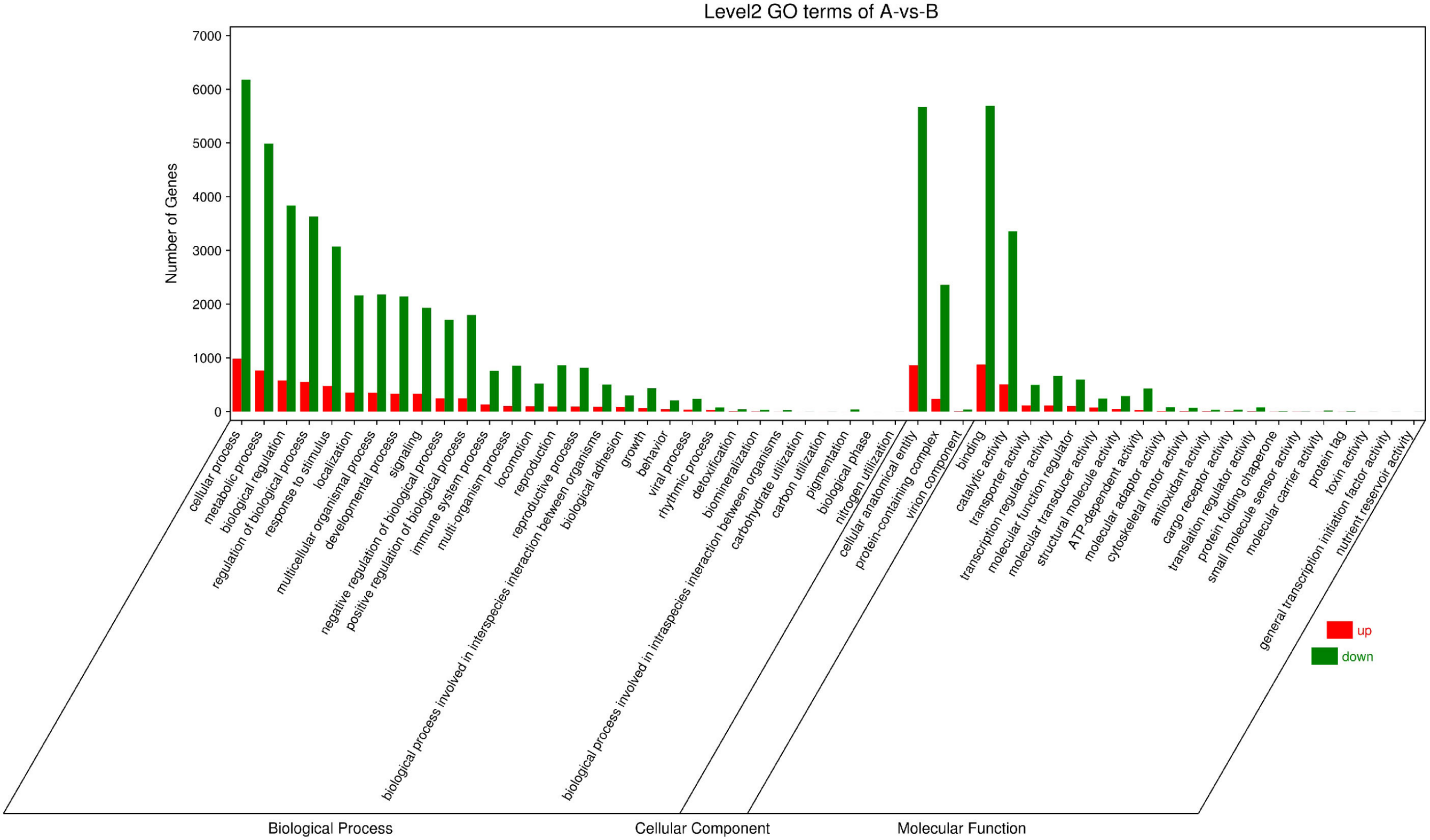
Column chart of GO enrichment classification. The abscissa is the secondary GOterm, and the ordinate is the number of differential genes in this term, with red indicating up-regulation and green indicating down-regulation.

The GO function annotation analysis was performed on the differentially expressed genes (**Figure 9**). What the consequences showed is that the identified differential genes were enriched in 30, 3 and 20 GO categories of biological process, cellular component, and molecular function accordingly. Differential genes related to biological processes are mainly involved in cellular processes, metabolic processes, biological regulation, regulation of biological processes, response to stimulation, and localization. The related differential genes in the cellular component are predominantly located in cellular anatomical entities and protein-containing complexes. Differential genes with molecular functions mainly affect binding and catalytic activity.

#### 3.4.3 KEGG enrichment analysis

**Figure 10:**
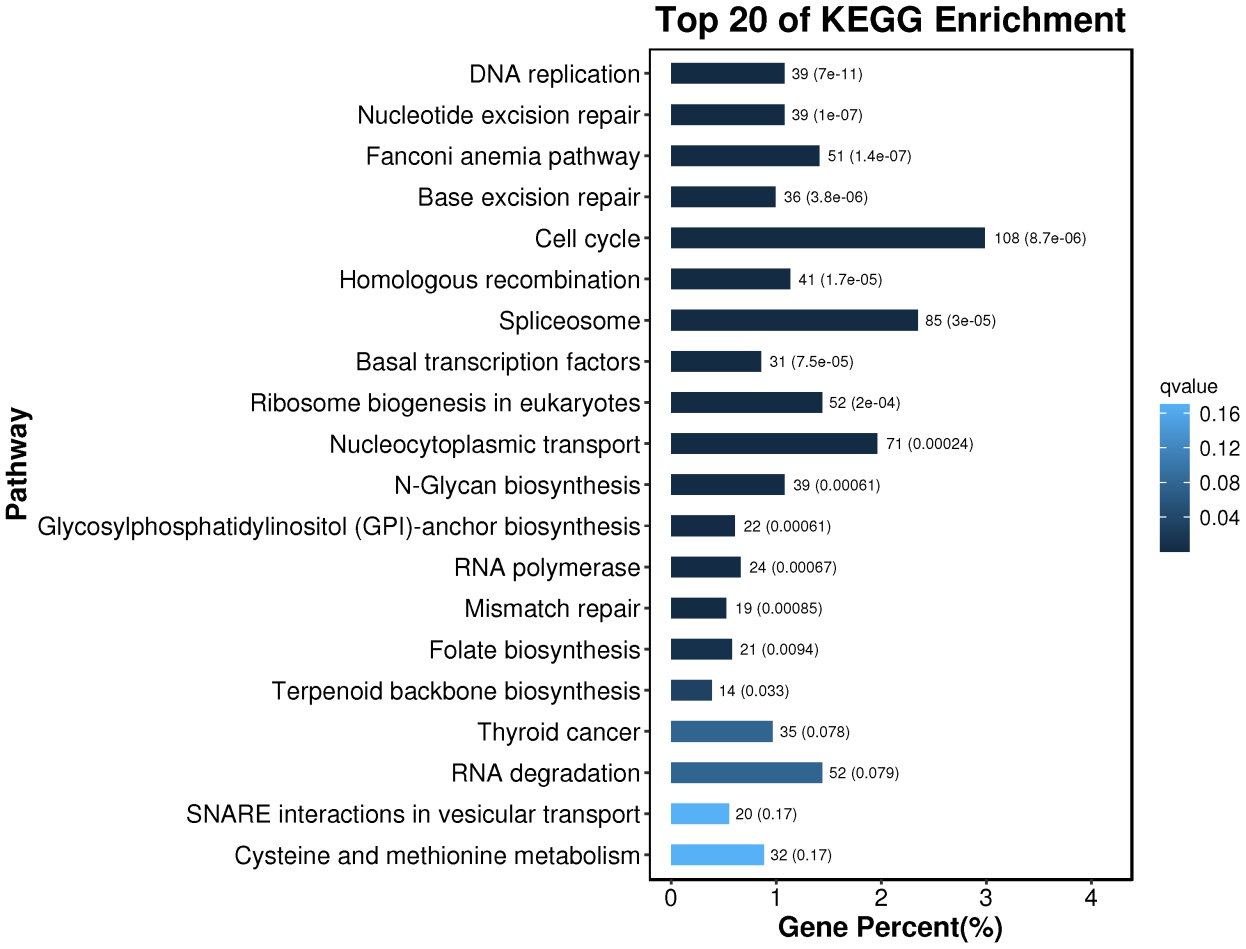
KEGG enrichment bar chart. The top 20 pathways with the smallest Q value are used for drawing. The ordinate is the pathway, and the abscissa is the percentage of the pathway number to all the different genes. The darker the color, the smaller the Q value, and the values on the column are the pathway number and Q value.

The obtained differential genes were enriched to 356 pathways by KEGG metabolic pathway analysis. According to the results of the KEGG enrichment differential bubble diagram, there exist noticeable differences in the pathways of DNA replication, Nucleotide Excessiveness Repair, Fanconinia pathway, Base Excessiveness Repair, Cell cycle, Homologous recombination, and Spliceosome in the treatment group of soybean fermentation powder, which is closely related to the improvement of the health status of loach by soybean fermentation powder. The 20 pathways, with the highest degree of differential gene enrichment, were analyzed. It was found that there were 108, 85 and 71 differential genes enriched in Cell cycle, Spliceosome, and Nucleocytoplasmic transport, respectively, which indicated that these three pathways played a significant role in improving the health status of Misgurnus anguillicaudatus by feeding fermented soybean powder (**Figure 10**).

### 3.5 qRT-PCR validation of DEG expression levels

**Figure 11:**
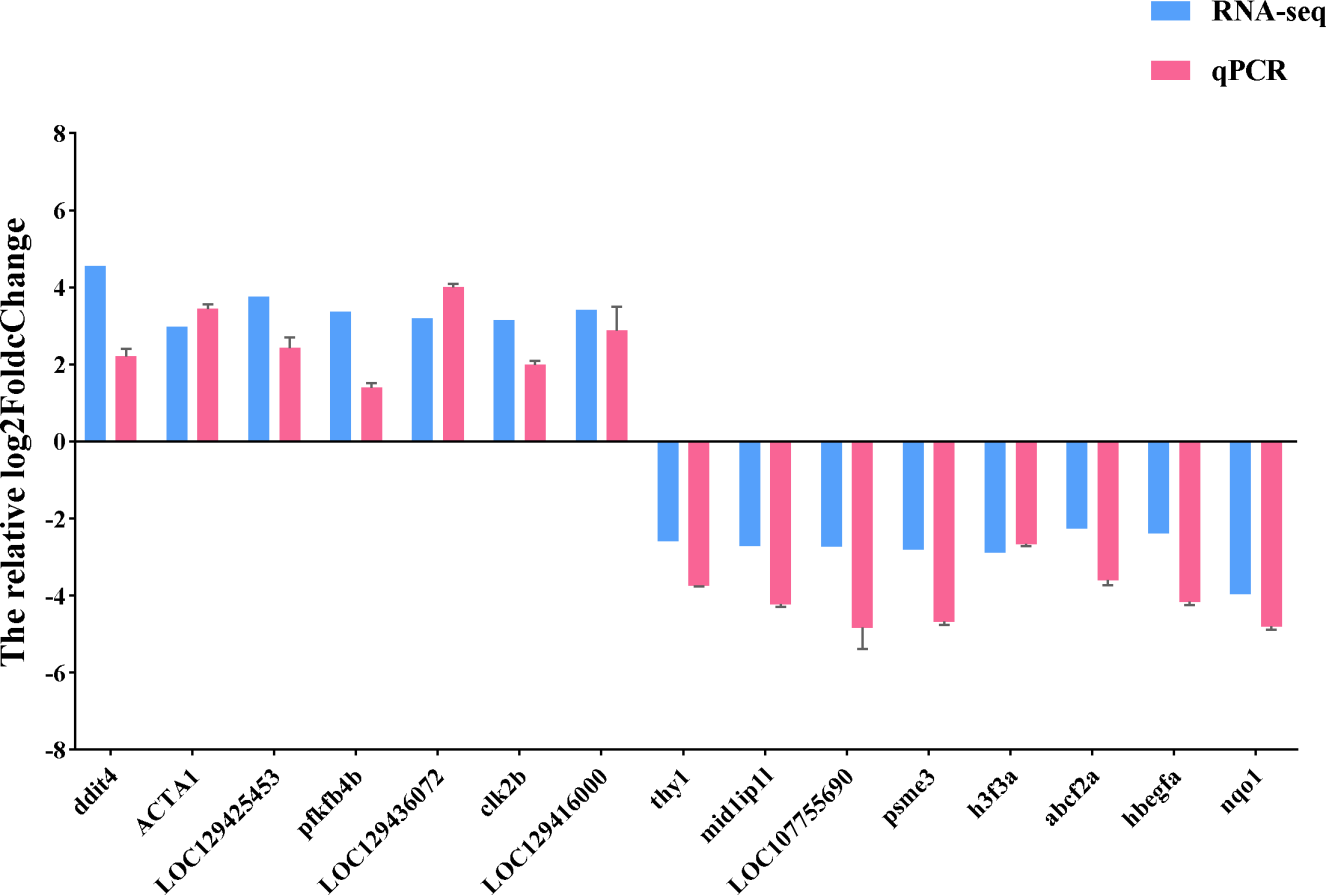
qRT-PCR validation of RNA-seq results. The relative log2FoldChange is shown by the ordinate, and the gene is represented by the abscissa. Genes that are up-regulated are above the horizontal line, while those that are down-regulated are below it.

**Figure 12:**
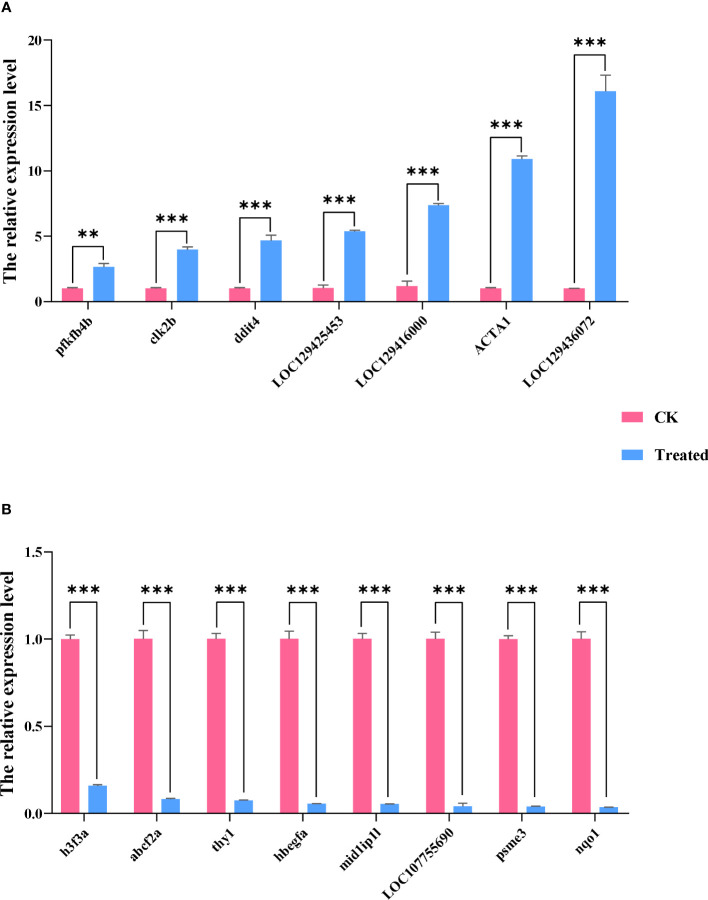
The qRT-PCR analysis of selected DEGs of the two groups. Data are presented as means ± SDs from three independent experiments. Student’s t-test was used for the statistical analysis between treated and untreated (control) samples in each rapeseed cultivar (**P < 0.01; ***P < 0.001). The abscissa represents the relative expression level, and the ordinate represents each gene. **(A)** shows up-regulated genes, and **(B)** shows down-regulated genes.

We used qRT-PCR to confirm the expression of 15 DEGs in the treatment group in order to validate the precision of RNA-seq in this investigation. All of the validated genes were expressed in a way that matched the sequencing data. Additionally, 7 up-regulated genes and 8 down-regulated genes were considerably up-regulated and down-regulated when compared to the control group, demonstrating the excellent dependability of the transcriptome data and the fact that it accurately captured transcriptome alterations (**Figures 11**, **12**).

## 4 Conclusion

In this study, 43,190,774 (99.57%) and 45,079,698 (99.30%) clean reads were attained by sequencing the control group (Group A) and the soybean fermented powder treatment group (Group B). The GC contents of clean reads obtained in Group A and Group B were 44.31% and 44.47% one by one, in the meanwhile, the Q20% was 97.14% and 96.46%, and the Q30% was 91.67% and 90.39% successively, which exhibited that the sequencing quality was high. We assembled reads and got 69,384 pieces of Unigene. At the same time, the gene functions of these Unigenes were annotated, and the Unigenes were compared to Nr, SwissProt, KEGG, and KOG databases by Blast. The NR database (43.58%) and KEGG database (42.33%) had the most annotations, while the KOG database (24.00%) had the poorest annotations.

With the purpose of exploring the mechanism of soybean fermentation powder in improving the immune function of loach, we further analyzed the differentially expressed genes between the two groups; and annotated the functions of the GO and KEGG database. The results of GO annotation showed that the differential genes related to biological processes are mainly involved in cellular processes, metabolic processes, and biological regulation. Meanwhile, the KEGG functional annotation results showed that there were 108, 85, and 71 differential genes enriched in three pathways: Cell cycle, Spliceosome, and Nucleocytoplasmic transport, respectively, which indicated that these three pathways played a critical role in improving the health status of loach by feeding loach with soy yeast powder.

In this study, based on transcriptome sequencing technology, the mechanism of soybean fermentation powder mediated by LPS on the immune function of the loach was explored, which indicated that soybean fermentation powder had a confident influence on the immune function of the loach. This study provides a new conception for the rational utilization of soybean fermented powder and theoretical guidance for the healthy feeding of loach in the later period.

## Data availability statement

The data presented in the study are deposited in the NCBI repository, accession numbers SRR25046463 and SRR25046464.

## Author contributions

JC and LD designed the research. YX, JC and LD performed the research. YX, XZ, DL, KQ, YL, TX analyzed the data. YX, DL, KQ, YL, JC and LD contributed reagents/materials/analysis tools. YX, XZ, JC and LD wrote the paper with other authors. All authors contributed to the article and approved the submitted version.
